# The association between caries experience and demographic, socioeconomic, and psychometric factors among persons with severe psychiatric and/or substance use disorders: a cross-sectional study

**DOI:** 10.2340/aos.v84.45205

**Published:** 2025-12-29

**Authors:** Kristina G. Kantola, Rolf Wynn, Jan-Are Kolset Johnsen, Elin Hadler-Olsen

**Affiliations:** a The Public Dental Health Service Competence Center of Northern Norway, Tromsø, Norway; b Department of Clinical Medicine, Faculty of Health Sciences, UiT The Arctic University of Norway, Tromsø, Norway; c Department of Clinical Dentistry, Faculty of Health Sciences, UiT The Arctic University of Norway, Tromsø, Norway; d Department of Medical Biology, Faculty of Health Sciences, UiT The Arctic University of Norway, Tromsø, Norway

**Keywords:** oral health, dental caries, DMFT index, substance use, psychiatric disorders

## Abstract

**Objective:**

This study examined caries experience and its association with demographic, socioeconomic, and psychometric factors among persons with severe psychiatric and/or substance use disorders.

**Material and methods:**

A cross-sectional study was conducted among inpatients at the Division of Mental Health and Substance Use, University Hospital of Northern Norway. Clinical oral examinations assessed decayed (D), missing (M), and filled (F) teeth separately and combined (DMFT). A questionnaire assessed demographic and socioeconomical factors, as well as variables from several health domains, including oral health. Analyses included descriptive statistics, cross-tabulations, and regression models.

**Results:**

The study included 136 adults (mean age 37.7 years, range 19–70, 60% men). Mean scores were decayed teeth (DT) = 4.6 standard deviation (SD = 4.8), filled teeth (FT) = 7.9 (SD = 5.3), missing teeth (MT) = 2.6 (SD = 4.4), and DMFT = 13.6 (SD = 7.8). About 80% had at least one decayed tooth; 45% had DT ≥ 4. Eight percent had fewer than 20 teeth. DT was significantly associated with age, substance use, and toothbrushing frequency. DMFT was associated with age and toothbrushing frequency.

**Conclusions:**

Findings reveal substantial unmet dental care needs, underscoring the importance of tailored interventions within the dental healthcare system.

## Background

Individuals with severe psychiatric disorders face significantly higher risks of comorbid somatic illness such as cardiovascular diseases, cancer, respiratory diseases, infections, and diabetes, which contribute to a reduced lifespan of 10–20 years compared to the general population [1–5]. This reduction in life expectancy is partly due to challenges in maintaining a healthy lifestyle, involving factors such as diet, smoking, alcohol use, and physical activity [6, 7]. Despite these challenges, individuals with severe psychiatric disorders and/or substance use disorders often encounter obstacles to their utilization of health services, resulting in suboptimal care [8–11].

Severe psychiatric disorders, such as schizophrenia and bipolar disorder, as well as substance use disorders, are also closely linked to poor oral health [12–17]. Individuals with severe psychiatric disorders generally have significantly more dental decay than the general population, and an increased risk of complete tooth loss [17]. Similarly, individuals with substance use disorders often exhibit an extensive need for dental treatment [18]. The high caries experience may be due to an irregular lifestyle with a poor diet, inadequate oral hygiene, and use of medications that can cause dry mouth and hyposalivation, increasing the risk of dental decay [19–22]. Low socioeconomic status is common in these groups, and is also associated with dental caries [23–26], and may limit access to necessary healthcare services. In Norway, adults normally pay for dental services out of pocket [27], making access sensitive to personal finances. A systematic review and meta-analysis [22] showed that individuals with severe mental illness were significantly less likely to visit the dentist and to brush their teeth regularly. Consequently, dental visits may be postponed until acute problems arise, when teeth may be too damaged to restore, leaving extraction as the only treatment option.

In Norway, large population studies typically include a broad segment of the general population. However, individuals with severe psychiatric disorders and/or substance use disorders rarely participate in these screenings. Consequently, this group remains understudied, leaving a considerable knowledge gap regarding their oral health status, associated challenges, and effective strategies to address their needs.

This issue is further exacerbated by the absence of a national registry or structured system to identify individuals with psychiatric, or substance use disorders, making it challenging to recruit participants for research. Many individuals in this vulnerable group, however, are periodically admitted to inpatient wards, offering a potential avenue for study.

The University Hospital of North Norway serves as the primary hospital for Northern Norway, admitting patients from the three counties of Finnmark, Troms, and Nordland. Focusing on participants admitted to this hospital provides access to a selected population that nevertheless represents diverse geographical areas.

This study aimed to determine the caries experience in a population with severe psychiatric disorders and/or substance use disorders and the association between caries experience and demographic, socioeconomic, and psychometric variables, providing insights into the factors contributing to oral health disparities in this vulnerable group.

## Methods

### Study design and recruitment

This cross-sectional study was conducted among inpatients at the University Hospital of North Norway, between October 2021 and July 2023. Participants answered a structured questionnaire and underwent a clinical oral examination.

Patients aged 18 and above, admitted to the Division of Mental Health and Substance Use at the University Hospital of Northern Norway, were eligible for participation. They were recruited from the following hospital units: the Emergency psychiatric wards, the Security wards, the Ward for patients with co-occurring substance use and severe mental illness, and the Substance use treatment wards. Patients younger than 18 years, patients admitted to other wards, and those who were deemed unable to provide informed consent were excluded from the study.

Eligible patients were informed about the study by ward staff, as well as through wall posters and table flyers displayed in the wards. Healthcare workers approached patients who appeared to meet the inclusion criteria, and those expressing interest received detailed written and oral information from dental assistants.

Before inclusion, participants gave written informed consent. Participants were offered complimentary dental cleanings and a 200NOK (approximately 17 Euros) gift card. Of the 138 patients that participated, two were excluded from the present study because they were edentulous.

### Procedures

The dentist performed clinical examinations in a dental chair, using a mouth mirror, three-way syringe and cotton rolls. The examination was supplemented by five to nine clinical photos (Camera: Nikon D7000, Lense: Nikon SWM VR ED IF Micro 1:1 d62 Nano Crystal Coat/AF-S Micro Nikkor 105 mm 1:2.8G ED), four Bitewing- and two apical radiographs. The assistants conducted structured interviews with the participants, with predetermined questions in a fixed format. Their training focused on presenting the questions neutrally and clarifying them as needed to prevent varied interpretations by the participants.

### Variables

#### Outcomes – caries experience

This study assessed the participants’ current and accumulated caries experience. The third molars were excluded from all analyses; thus 28 teeth were regarded as a full set of teeth. Caries, restorations and missing teeth (MT) were registered based on clinical examinations, radiographs and clinical photographs.

***Decayed teeth (DT)*** (primary and secondary) were recorded and scored from 1 to 5 based on visual inspection supported by clinical photographs and radiographs. Grade 1 and 2 indicated caries in the outer and inner half of enamel, respectively, and grade 3, 4 and 5 indicated caries extending into the outer third, middle and inner third of the dentin, respectively. This paper defines caries (DT) as grade 3–5, manifest caries reaching the dentine.

***Missing teeth (MT)*** were registered regardless of cause of loss.

***Filled teeth (FT)*** included teeth with all types of permanent restorations, including crowns and bridge abutments.

***The DMFT index*** was calculated by summarizing the number of DT, MT, and FT in an individual, with a range of 0–28.

#### Quality controls

Consistency within radiography procedures was maintained with all images captured using the same machine and settings (Planmeca Intra, 70kV, 0.16 sec), following pre-checks to ensure compliance with standards. All radiographs were analyzed in a consistent and controlled environment, free from external light interference, and on the same computer screen.

To ensure consistency in caries registration across patients and over time, the dentist performing all clinical examinations and post-registrations, re-evaluated every 15th case 1 month after completion of the last participant, under the same conditions as the initial assessment. Intraclass Correlation Coefficient (ICC) was calculated with a two-way mixed-effects model with absolute agreement to assess intra-observer agreement on a 10-case dataset. The intra-rater reliability for primary and secondary caries registration was good (ICC = 0.875, 95% CI: 0.862–0.888 and ICC = 0.783, 95% CI: 0.759–0.805, respectively).

#### Explanatory variables

Explanatory variables were derived from the questionnaire and included the following: sex, age, education, work situation, financial situation, tobacco use, substance use, alcohol consumption, psychological distress, prodromal symptoms, medications, xerostomia, and dental attendance. Details of the questions, response options, and their reclassification for analysis are provided in Table 1.

**Table 1:** Explanatory variables.

Variables	Question/Psychometric instrument	Options in questionnaire	Recoded for	
			Descriptive analysis	Regression
Sex		Female/Male	Female/Male	Female/Male
Age		Derived from birth year	19–29 years 30–39 years 40–49 years ≥ 50 years	19–29 years 30–49 years ≥ 50 years
Education	What is your highest level of completed education?	1. Elementary school 2. High School or vocational education 3. College/university less than 4 years 4. College/university 4 years or more	1. Elementary school 2. High School/vocational education 3. College/University (original options 3 and 4)	1. Elementary school 2. High School/vocational education 3. College/University (originals option 3 and 4)
Employment	What is your current work or life situation? (multiple answers possible)	1. Work full-time 2. Work part-time 3. Student 4. Maternity leave 5. Sick leave 6. Unemployed 7. Receiving Work Assessment Allowance (AAP) 8. Receiving disability benefits 9. Receiving social benefits	1. Within the workforce (fulltime, part-time work or student (original options 1, 2 and 3) 2. Outside workforce (original options 4, 5, 6, 7, 8 and 9)	Not included in regression
Financial situation	How would you rate your current financial situation?	1. Very good 2. Good 3. Average 4. Difficult 5. Very Difficult	1. Good (original options 1 and 2) 2. Average (original option 3) 3. Difficult (original options 4 and 5)	1. Good/average (original options 1, 2 and 3) 2. Difficult (original options 4 and 5)
Tobacco	Two separate questions: Do you smoke? Do you use snuff?	1. No, never 2. Before, but not anymore 3. Sometimes 4. Daily	1. No, never 2. Before, but not any more 3. ‘Current smoker’, or ‘current snuff user’ (original options 3 and 4)	Not included in regression
Regular dental visits	Do you visit a dentist or dental hygienist regularly?	1. More than once a year 2. Once a year 3. Every second year 4. Regularly but less than every second year 5. Only for acute problems 6. Never	1. At least every second year (options 1, 2, and 3) 2. Less than every second year (options 4, 5, and 6)	1. At least every second year (options 1, 2, and 3) 2. Less than every second year (options 4, 5, and 6)
Tooth brushing frequency	How often do you brush your teeth?	1. Less than once a week 2. A few times a week 3. Once a day 4. Twice a day or more	1. Less than daily (option 1 and 2) 2. Once a day 3. Twice a day or more	1. Less than once a day (option 1 and 2) 2. Once a day or more
Alcohol consumption*	Audit-C How often do you drink alcohol?		1. Low 2. Moderate 3. High	Not included in regression
Substance use	Do you use any other substances or addictive medications? Type of drugs was registered as free text.	1. Never 2. Have tried sometime 3. Once a month or less 4. 2–4 times a month 5. 2–3 times a week 6. 4 times a week or more 7. Check if you mean during relapses, or in certain periods	1. Never (original option 1) 2. 1–4 times a month (original option 2, 3 and 4) 3. More than 4 times a month/relapses (original options 5, 6 and 7)	1. Never (original option 1) 2. Sometimes/Often (original option 2, 3, 4, 5, 6, 7)
Anxiety/Depression**	Hopkins Symptom Check List-10 (HSCL)		1. No/low distress (≤ 2.35) 2. Moderate/high distress (> 2.35)	1. No/low distress (≤ 2.35) 2. Moderate/high distress (> 2.35)
Prodromal symptoms/ Psychosis risk ***	The 16-item Version of the Prodromal Questionnaire (PQ-16)		1. 0–5 symptoms 2. ≥ 6 symptoms	1. 0–5 symptoms 2. ≥ 6 symptoms
Prescribed medications	Yes/No Type of medications in free text	1. No medications 2. 1–2 prescribed medications 3. More than two prescribed medications	1. No medications 2. 1–2 prescribed medications 3. More than two prescribed medications	Not included in regression
Xerostomia	Do you feel bothered by dry mouth?	1. Not at all 2. A little bothered 3. Quite bothered 4. Very bothered	1. No/little feeling of dry mouth (original option 1 and 2) 2. Moderate/strong feeling of dry mouth (original option 3 and 4)	1. No/little feeling of dry mouth (original option 1 and 2) 2. Moderate/strong feeling of dry mouth (original option 3 and 4)

* *Audit C:* The validated Audit-C screening tool was used to identify problematic alcohol use [28]. Participants reported both the frequency of alcohol intake and average number of units consumed over the past 12 months. Max score was 12. For men, a cut off at 4 or more indicates harmful use or dependence, while for women a cut off of 3 or more indicates harmful use or dependence [28–31]. However, in this study, which included a sample of inpatients with a presumed overall higher use of alcohol, the decision was made to separate into three categories: Low (Audit-C = 0–3), Moderate (Audit-C = 4–7), and High (Audit-C = 8–12) for both men and women.

** *Anxiety/Depression: Hopkins Symptom Check List-10 (HSCL):* Symptoms of anxiety and depression were assessed with the Hopkins Symptom Check List-10 (HSCL-10, consisting of 10 validated questions [32, 33]. The average score was calculated and dichotomized into the categories No/low distress (≤ 2.35) and Moderate/high distress (> 2.35) [33].

*** *Prodromal symptoms/Psychosis risk: The 16-item Version of the Prodromal Questionnaire (PQ-16):* The PQ-16, which consists of 16 validated questions, was used for recording symptoms indicating a risk of developing or currently experiencing psychosis. In our study, responses were dichotomized: (1) 0-5 symptoms, and (2) 6 or more symptoms, in line with the recommendations for the (general) help-seeking population [34, 35].

### Statistical analysis

Statistical analyses were performed using IBM SPSS Statistics version 29.0 for Windows. A *p*-value < 0.05 was considered statistically significant.

Means with standard deviations (SD) and medians with 25 and 75 percentiles (Q1, Q3) were computed for DT, MT, FT and DMFT by sample characteristics. As the outcomes were skewed, distribution across variable categories was assessed with non-parametric tests (Mann–Whitney U-test and Kruskal–Wallis test).

Associations between DT, DMFT and explanatory variables were assessed using regressions. For DT, negative binomial regression was used to estimate unadjusted and adjusted rate ratios (RR) due to right-skewed distribution and indications of overdispersion. Model fit was evaluated using Deviance/df, Pearson Chi-Square/df, and Akaike’s Information Criterion (AIC).

For DMFT, linear regression was applied as key assumptions such as normally distributed residuals, linearity, and homoscedasticity were largely met. The explanatory variables for regressions were selected based on theoretical relevance, empirical findings, and clinical considerations and included sociodemographic variables such as age, gender, education [23, 26, 36], behavioral factors, such as oral hygiene habits, dental visits and substance use [18, 37], psychological variables [15, 17] and clinical variables, including self-reported xerostomia [38, 39].

Multicollinearity among the independent variables was assessed using Variance Inflation Factor (VIF), condition indices, and variance proportions. The highest VIF observed was 2.9, indicating a low risk of multicollinearity. Although some condition indices exceeded 10, variance proportions did not reveal substantial clustering (>50%) between predictors on high condition index dimensions. Therefore, multicollinearity was not considered a major issue in the regression models.

For the linear regression of DMFT, the independence of residuals was assessed using the Durbin–Watson statistics. Outliers and influential cases were evaluated using standardized residuals, Cook’s distance, and leverage values.

Missing data: Data were missing for 0.7–2.9% of the participants for all variables, except the use of prescribed medication where the fraction of missing data was 4.4%. Missing data were excluded from analyses.

### Ethics

The study was approved by the Regional Committee for Medical and Health Research Ethics (240987) and by the Norwegian Centre for Research Data (119768). All participants gave written informed consent.

## Results

### Characteristics of the study population

Participants’ characteristics are described in Table 2. The mean age of the 136 participants was 37.7 years (range: 19–70 years), and approximately 60% were men (Table 2). The vast majority of the participants had not completed higher education, were outside the workforce and reported an average or difficult financial situation. Most of the participants reported tobacco use, with many being current smokers and an even larger proportion using snuff. Regular use of multiple prescribed medications was frequently reported and symptoms of dry mouth (Xerostomia) were also common. Dental care habits varied; few participants reported attending regular check-ups, and just over half brushed their teeth at least twice a day (Table 2).

**Table 2:** Distribution of decayed, missing, and filled teeth by age, sociodemographic, substance use, medications, distress, xerostomia, and oral care.

Variables	All *n* (%)	DT Mean (SD)	DT Median (Q1, Q3)	*p*	FT Mean (SD)	FT Median (Q1, Q3)	*p*	MT Mean (SD)	MT Median (Q1, Q3)	*p*	DMFT Mean (SD)	DMFT Median (Q1, Q3)	*p*
**All**		4.6 (4.8)	3.0 (1.0, 7.0)		7.9 (5.3)	7.0 (4.0, 11.0)		2.6 (4.4)	1.0 (0.0, 4.0)		13.6 (7.8)	13.0 (7.3, 20.0)	
**Sex**				0.514			0.797			0.216			0.497
Female	55 (40.4)	4.5 (5.2)	2.0 (1.0, 7.0)		7.9 (5.3)	7.0 (4.0, 11.0)		2.2 (3.9)	0.0 (0.0, 3.0)		13.1 (7.4)	12.0 (8.0, 19.0)	
Male	81 (59.6)	4.7 (4.6)	3.0 (1.0, 7.5)		7.8 (4.5)	7.0 (4.0, 11.0)		2.8 (4.7)	1.0 (1.0, 4.0)		13.9 (8.0)	13.0 (7.0, 20.5)	
**Age group**				0.184			< 0.001**			< 0.001***			< 0.001***
19–29	52 (38.2)	3.9 (4.7)	2.5 (0.3, 6.0)		5.4 (4.0)	5.0 (2.0, 8.0)		0.7 (1.8)	0.0 (0.0, 0.0)		9.4 (6.8)	8.5 (4.0, 13.0)	
30–39	27 (19.9)	4.6 (4.0)	3.0 (1.0, 8.0)		8.9 (4.5)	8.0 (6.0, 12.0)		2.6 (3.3)	1.0 (0.0, 4.0)		13.5 (6.6)	12.0 (9.0, 17.0)	
40–49	32 (23.5)	6.2 (5.8)	5.0 (2.0, 8.8)	0.035*	8.3 (5.2)	7.0 (5.0, 11.0)		3.6 (5.3)	1.5 (1.0, 4.0)		16.2 (7.3)	15.5 (10.5, 22.8)	
≥ 50	25 (18.4)	4.0 (4.1)	3.0 (0.50, 6.5)		11.7 (6.1)	11.0 (6.5, 15.0)		5.2 (6.1)	3.0 (1.0, 6.5)		19.1 (6.8)	21.0 (12.5, 25.0)	
**Substance use**				0.001****			0.717			0.997			0.842
Never	45 (33.6)	3.0 (4.8)	1.0 (0.0, 4.0)		8.6 (6.2)	7.0 (4.0, 12.5)		2.9 (5.2)	1.0 (0.0, 2.5)		13.5 (9.4)	12.0 (5.5, 23.0)	
1–4/month	25 (18.7)	5.4 (4.3)	5.0 (1.5, 8.5)		8.6 (6.2)	7.0 (4.5, 13.0)		2.2 (3.2)	1.0 (0.0, 3.5)		14.0 (7.1)	15.0 (7.5, 19.5)	
> 4/month	64 (47.8)	5.1 (4.5)	3.5 (2.0, 8.0)		7.3 (4.4)	7.0 (4.3, 9.0)		2.4 (4.2)	0.5 (0.0, 4.0)		13.3 (6.8)	12.0 (9.0, 18.0)	
**Alcohol consumption**				0.096			0.279			0.120			0.557
Low	57 (43.2)	3.7 (4.1)	3.0 (0.0, 6.0)		8.4 (5.2)	8.0 (5.0, 12.0)		3.3 (5.2)	1.0 (0.0, 4.0)		14.2 (8.1)	13.0 (8.0, 20.5)	
Moderate	34 (25.8)	5.2 (5.2)	3.0 (1.0, 9.0)		7.9 (4.9)	7.0 (4.0, 9.3)		1.6 (3.2)	0.0 (0.0, 1.0)		12.4 (7.3)	12.0 (7.0, 17.3)	
High	41 (31.1)	5.0 (4.9)	3.0 (1.0, 7.0)		7.4 (5.7)	6.0 (3.0, 10.5)		2.5 (4.0)	1.0 (0.0, 4.0)		13.8 (7.7)	14.0 (7.0, 21.0)	
**Psychological distress**				0.201			0.510			0.295			0.546
No/low ≤ 2.35	72 (54.1)	4.1 (4.2)	3.0 (1.0, 6.0)		7.5 (5.4)	6.0 (4.3, 9.8)		2.8 (4.4)	1.0 (0.0, 4.0)		13.1 (7.7)	12.0 (7.3, 18.0)	
Moderate/high > 2.35	61 (45.9)	4.9 (5.1)	3.0 (1.0, 7.0)		8.4 (5.1)	9.0 (4.0, 12.0)		2.3 (4.4)	0.0 (0.0, 3.0)		13.9 (7.9)	13.9 (7.0, 21.0)	
**Psychosis risk**				0.541			0.109			0.696			0.309
0–5 symptoms	81 (60.9)	4.5 (4.3)	3.0 (1.0, 7.0)		8.4 (5.1)	8.0 (5.0, 11.5)		2.7 (4.4)	1.0 (0.0, 3.5)		14.0 (7.4)	13.0 (8.5, 20.0)	
≥ 6 symptoms	52 (39.1)	4.5 (5.2)	2.5 (1.0, 7.0)		7.1 (5.6)	6.0 (3.3, 9.8)		2.4 (4.4)	0.0 (0.0, 4.0)		12.8 (8.4)	11.0 (6.0, 18.8)	
**Education**				**0.023**			**0.044**			0.834			0.682
Elementary	48 (36.4)	**5.3** **(4.5)**	**4.5** **(2.0, 8.0)**		**6.6** **(4.8)**	**6.0** **(3.0, 9.0)**		3.2 (5.0)	0.5 (0.0, 4.8)		13.4 (8.1)	12.5 (7.0, 18.0)	
High school/vocational	67 (50.8)	4.4 (4.9)	3.0 (1.0, 7.0)		8.5 (5.5)	8.0 (5.0, 11.0)		1.9 (2.8)	1.0 (0.0, 3.0)		13.2 (7.8)	13.0 (7.0, 21.0)	
College/University	17 (12.9)	**2.7** **(4.4)**	**1.0** **(0.0, 4.5)**		**9.9** **(5.6)**	**9.0** **(6.0, 15.5)**		3.7 (7.1)	1.0 (3.0, 4.5)		15.2 (7.7)	13.0 (8.0, 21.5)	
**Employment**				0.086			0.685			0.750			0.490
Part/fulltime employed or student	16 (11.9)	2.6 (3.1)	1.5 (0.0, 4.8)		8.4 (5.2)	7.0 (4.3, 10.8)		2.2 (3.3)	0.0 (0.0, 4.5)		12.4 (8.6)	10.0 (6.0, 23.0)	
Outside workforce	119 (88.1)	4.8 (4.8)	3.0 (1.0, 7.0)		7.9 (5.3)	7.0 (4.0, 11.0)		2.6 (4.5)	1.0 (0.0, 4.0)		13.7 (7.7)	13.0 (8.0, 19.5)	
**Financial situation**				**0.037**			0.534			0.695			0.684
Good	32 (24.2)	**2.8** **(3.0)**	**2.0** **(0.0, 4.8)**		8.8 (6.0)	7.5 (4.3, 11.8)		2.6 (2.8)	0.0 (0.0, 3.5)		12.8 (7.5)	12.5 (7.0, 18.5)	
Average	51 (38.6)	4.6 (4.9)	3.0 (1.0, 6.0)		8.2 (4.5)	7.0 (5.0, 11.0)		3.2 (5.1)	1.0 (0.0, 4.0)		14.2 (7.6)	12.0 (9.0, 19.0)	
Difficult	49 (37.1)	**5.7** **(5.2)**	**4.0** **(2.0, 9.0)**		7.4 (5.6)	7.0 (2.5, 10.5)		2.7 (4.7)	1.0 (0.0, 3.5)		14.0 (8.0)	13.0 (6.5, 21.5)	
**Smoking**				**0.009**			0.112			0.235			**0.031**
Never	18 (13.2)	**1.9** **(2.5)**	**1.0** **(0.0, 4.0)**		6.5 (4.3)	6.0 (4.0, 8.3)		1.0 (1.4)	0.0 (0.0, 2.0)		**9.1** **(5.4)**	**8.5** **(5.5, 11.5)**	
Previous	43 (31.6)	4.2 (4.4)	3.0 (1.0, 7.0)		8.9 (4.7)	9.0 (6.0 13.0)		2.3 (3.7)	0.0 (0.0, 3.0)		14.0 (7.3)	13.0 (8.0, 18.0)	
Current	74 (54.4)	**5.3** **(5.0)**	**3.0** **(2.0, 8.0)**		7.8 (5.8)	6.0 (3.8, 11.0)		3.1 (5.3)	1.0 (0.0, 4.0)		**14.4** **(8.2)**	**13.0** **(7.0, 22.0)**	
**Snuff**				0.939			0.146			0.321			0.252
Never	37 (27.6)	4.7 (4.9)	4.0 (0.5, 7.0)		8.9 (6.0)	7.0 (5.0–13.0)		3.4 (5.2)	2.0 (0.0–4.0)		15.1 (8.2)	15.0 (9.5, 22.5)	
Previous	7 (5.2)	4.6 (4.4)	3.0 (1.0, 10.0)		10.6 (5.7)	10.0 (5.0–16.0)		2.6 (4.2)	0.0 (0.0–5.0)		14.7 (8.0)	12.0 (9.0, 23.0)	
Current	90 (67.2)	4.4 (4.7)	3.0 (1.0, 7.0)		7.3 (4.9)	6.5 (4.0–9.3)		2.1 (3.9)	0.0 (0.0–3.0)		12.7 (7.5)	12.0 (7.0, 17.3)	
**Medications**				0.128			0.393			0.812			0.141
0	12 (9.2)	2.4 (2.8)	1.0 (0.0, 4.8)		7.7 (5.5)	6.5 (4.5, 10.5)		1.8 (2.0)	1.5 (0.0, 3.8)		11.4 (7.3)	12.5 (5.5, 14.8)	
1–2 drugs	50 (38.5)	5.1 (4.7)	4.0 (1.0, 8.3)		8.7 (5.3)	8.5 (5.0, 12.5)		3.4 (5.2)	0.0 (0.0, 4.5)		15.2 (7.8)	16.0 (11.0, 21.3)	
≥ 3 drugs	68 (52.3)	4.5 (5.0)	3.0 (0.3, 7.0)		7.5 (5.3)	7.0 (4.0, 10.0)		2.3 (3.9)	2.3 (0.0, 2.0)		12.8 (7.5)	11.0 (7.0, 18.8)	
**Xerostomia**				0.503			0.247			0.307			0.364
No/little	91 (67.4)	4.6 (5.1)	3.0 (0.0, 7.0)		7.7 (5.5)	6.0 (4.0, 11.0)		8.4 (4.9)	0.0 (0.0, 4.0)		13.1 (8.0)	13.0 (6.0, 19.0)	
Moderate/severe	44 (32.6)	4.4 (3.9)	3.0 (1.0, 6.8)		8.4 (4.9)	8.0 (5.3, 11.8)		3.0 (4.2)	1.0 (0.0, 4.8)		14.4 (7.4)	13.0 (9.0, 21.0)	
**Tooth brushing frequency**				0.229			0.071			0.585			0.821
< 1/day	32 (23.7)	6.1 (6.1)	4.5 (1.0, 11.0)		**6.4** **(5.3)**	**6.0** **(2.0, 8.8)**	**0.023**	3.6 (6.4)	1.0 (0.0, 4.0)		14.3 (9.2)	13.0 (6.5, 24.5)	
1/day	31 (23.0)	4.4 (3.9)	3.0 (1.0, 7.0)		8.0 (5.5)	6.0 (5.0, 12.0)		2.8 (4.3)	1.0 (0.0, 4.0)		13.8 (7.7)	13.0 (7.0, 21.0)	
≥ 2/day	72 (53.3)	3.8 (4.1)	3.0 (0.3, 5.8)		**8.7** **(5.2)**	**9.0** **(5.0, 11.0)**		2.0 (3.2)	0.0 (0.0, 3.0)		13.1 (7.1)	12.5 (7.3, 18.0)	
**Regular dental visits**				**0.007**			0.593			0.902			0.891
At least every second year or more	39 (29.1)	**3.0** **(4.1)**	**2.0** **(0.0, 4.0)**		7.6 (5.9)	7.0 (3.0, 10.0)		3.1 (5.9)	1.0 (0.0, 3.0)		13.6 (8.1)	12.0 (8.0, 21.0)	
More than 2 years, acute, never	95 (70.9)	**5.3** **(5.0)**	**4.0** **(1.0, 8.0)**		7.9 (4.8)	7.0 (5.0, 11.0)		2.4 (3.6)	1.0 (0.0, 4.0)		13.4 (7.7)	13.0 (7.0, 19.0)	

Numbers in bold indicate two-sided *p*-value <0.05 between groups.

* *p* = 0.035 between age group 20–29 and 40–49 years;

** *p* < 0.001 between age group 19–29 years and all other age groups;

*** *p* < 0.001 between all age groups;

**** *p* < 0.05 between substance group ‘never’ and 1–4/month, and between substance group ‘never’ and > 4/month.

*n*: number in the group; Q1, Q3: 25th percentile, 75th percentile; DT: decayed teeth; FT: filled teeth; MT: missing teeth; DMFT: decayed, missing and filled teeth; SD: standard deviation; Perc: percentile; *p*: 95% confidence interval, two-sided *p*-value.

The participants were inpatients at hospital units treating substance use disorders and/or psychiatric illness. Accordingly, high alcohol consumption, frequent substance use, and moderate to high levels of emotional distress were common. Prodromal symptoms, associated with psychosis risk, were also reported by a notable portion of the participants.

### Decayed teeth

The prevalence of dentine caries was 79.4%, where 34.6% had 1–3 DT, and 44.9% had four or more DT (Table 3). The mean number of DT was 4.6 (SD = 4.8) and was highest in the 40–49-year age-group and lowest in the 19–29-year age-group (Table 2). Relatively high numbers of DT were found among participants with elementary school as their highest level of education, those reporting a difficult financial situation, cigarette smokers, participants with infrequent dental visits and those who used substances either occasionally or frequently (Table 2).

**Table 3:** Distribution of decayed and remaining teeth by sex and age group.

	All *n*	DT			Remaining teeth	
		DT = 0 *n* (%)	DT = 1–3 *n* (%)	DT ≥ 4 *n* (%)	≥ 20 *n* (%)	< 20 *n* (%)
All	136	28 (20.6)	47 (34.6)	61 (44.8)	125 (91.9)	11 (8.1)
Sex						
Female	55	12 (21.8)	20 (36.4)	23 (41.8)	51 (92.7)	4 (7.3)
Male	81	16 (19.8)	27 (33.3)	38 (46.9)	74 (91.4)	7 (8.6)
Age group						
19–29	52	13 (25.0)	21 (40.4)	18 (34.6)	52 (100.0)	0 (0.0)
30–39	27	4 (14.8)	10 (37.0)	13 (48.1)	25 (92.6)	2 (7.4)
40–49	32	5 (15.6)	8 (25.0)	19 (59.4)	28 (87.5)	4 (12.5)
≥ 50	25	6 (24.0)	8 (32.0)	11 (44.0)	20 (80.0)	5 (20.0)

*n*: number in the group; DT: decayed teeth.

#### Negative binominal regression (DT)

Univariate and multivariate data from the regression analysis are presented in Table 4. In the adjusted model, age, substance use, and tooth brushing frequency were significantly associated with DT. Participants aged 30–49 years had a 63% higher rate of DT compared to those aged 19–29, and individuals who reported occasional or frequent substance use had a 73% higher rate of DT than non-users. In addition, compared to brushing teeth at least once a day, brushing less than once daily was associated with a 70% higher rate of DT.

**Table 4:** Negative binominal regression – outcome variable DT.

Variable	Unadjusted IRR (95% CI)	Adjusted IRR (95% CI)
Sex		
Female	Reference	Reference
Male	1.04 (0.71, 1.51)	0.94 (0.62, 1.43)
Age group		
19–29 years	Reference	Reference
30–49 years	1.40 (0.93, 2.12)	1.63 (1.02, 2.60)*
50+ years	1.04 (0.61, 1.77)	1.17 (0.64, 2.16)
Education		
Elementary	2.00 (1.06, 3.78)**	2.04 (0.99, 4.21)
High School/Vocational	1.67 (0.90, 3.10)	1.86 (0.94, 3.67)
College/University	Reference	Reference
Financial situation		
Good/average	Reference	Reference
Difficult	1.46 (0.99, 2.16)	1.38 (0.90, 2.12)
Psychological distress		
≤ 2.35	Reference	Reference
> 2.35	1.19 (0.82, 1.74)	1.24 (0.77, 1.97)
Psychosis risk		
0–5 symptoms	Reference	Reference
≥ 6 symptoms	1.00 (0.68, 1.47)	0.79 (0.51, 1.23)
Xerostomia		
No/little/moderate	Reference	Reference
Severe	0.96 (0.64, 1.43)	0.82 (0.53, 1.28)
Substance use		
Never	Reference	Reference
Sometimes/often	1.75 (1.16, 2.63)***	1.73 (1.08, 2.77)****
Toothbrushing frequency		
< 1/day	1.52 (0.99, 2.35)	1.70 (1.04, 2.80)*****
Daily or more	Reference	Reference
Regular dental visits		
At least every second year or more	Reference	Reference
More than 2 years, acute, never	1.14 (0.88, 2.03)	1.16 (0.72, 1.88)

Bold values indicate IRRs that differ significantly from the reference category (p < 0.05).

* *p* = 0.041,

** *p* = 0.033,

*** *p* = 0.007,

**** *p* = 0.024,

***** *p* = 0.035.

IRR indicates the relative rate of the outcome in one group compared to the reference group. IRR: incidence rate ratio; CI: confidence interval; DT: decayed teeth.

The regression model for DT showed goodness-of-fit statistics indicating an adequate model fit, with a Deviance/df of 1.07 and a Pearson Chi-Square/df of 0.93. The AIC was 666.2. No violations of distributional assumptions were detected. The full model significantly outperformed the intercept-only model.

### Filled teeth

The mean number of FT was 7.9 (SD = 5.3). Relatively high numbers of FT were found among the older age groups, those who had completed higher education and those who brushed their teeth at least twice daily (Table 2).

### Missing teeth

The mean number of MT was 2.6 (SD = 4.4). Overall, 8% of participants had fewer than 20 remaining teeth. In the age groups 40–49 years and 50 years or older 12.5 and 20.0%, respectively, had fewer than 20 teeth (Table 3). The number of MT was significantly lower in the youngest age group compared to the older age groups (Table 2). No significant differences in MT were observed between categories of any other co-variates (Table 2).

### Accumulated caries experience (DMFT)

The mean DMFT score was 13.6 (SD = 7.8). Only 2.2% of the participants had no caries experience, while the majority had extensive caries experience. The DMFT score was relatively high among current smokers and participants in the older age groups (Table 2).

#### Linear regression (DMFT)

Results from univariate and multivariate linear regressions with DMFT as outcome are presented in Table 5.

**Table 5:** Linear regression – outcome variable DMFT.

Variable	Unadjusted Unstandardized B (95% CI)	Adjusted Unstandardized B (95% CI)
Sex		
Female (reference)	Reference	Reference
Male	0.87 (-1.82, –3.55)	1.24 (-1.37, 3.86)
Age group		
19–29 years	Reference	Reference
30–49 years	5.58 (2.99, 8.18)*	6.15 (3.31, 8.99)*
50+ years	9.70 (6.38, 13.02)*	11.15 (7.33, 14.98)*
Education		
Elementary	-1.76 (-6.15, 2.63)	2.22 (-2.05, 6.50)
High School/Vocational	-1.94 (-6.16, 2.29)	0.61 (-3.34, 4.56)
College/University	Reference	Reference
Financial situation		
Good/average	Reference	Reference
Difficult	0.34 (-2.41, 3.09)	0.45 (-2.21, 3.10)
Psychological distress		
≤ 2.35	Reference	Reference
> 2.35	0.81 (-1.87, 3.49)	1.47 (-1.34, 4.28)
Psychosis risk		
0–5 symptoms	Reference	Reference
≥ 6 symptoms	-1.23 (-3.95, 1.50)	-1.16 (-3.94, 1.62)
Xerostomia		
No/little/moderate	Reference	Reference
Severe	1.29 (-1.53, 4.11)	1.45 (-1.26, 4.15)
Substance use		
Never	Reference	Reference
Sometimes/often	-0.39 (-2.85, 2.77)	0.54 (-2.29, 3.8)
Toothbrushing frequency		
< 1/day	1.05 (-2.06, 4.17)	3.27 (0.20, 6.33)**
Daily or more	Reference	Reference
Regular dental visits		
At least every second year or more	Reference	Reference
More than 2 years, acute, never	0.13 (-2.84, 3.10)	-0.40 (-3.19, 2.40)

Bold values indicate IRRs that differ significantly from the reference category (p < 0.05).

* *p* < 0.001,

** *p* = 0.037.

B: Unstandardized B shows the change in DMFT compared to the reference value, CI: confidence interval; DMFT: decayed, missing, filled teeth index.

Among the predictors, age showed the strongest association with DMFT. Participants aged 30–49 years and 50 and above had significantly higher DMFT scores, with an unstandardized B of 6.15 and 11.15, respectively. Toothbrushing frequency was also significantly associated with DMFT in the adjusted model showing that those brushing less than once a day had significantly higher DMFT than those brushing daily or more, and an unstandardized B of 2.27.

The Durbin–Watson statistic was 2.06. No standardized residuals exceeded ±3, the maximum Cook’s distance was 0.06 and the highest leverage value was 0.13. The linear regression model was statistically significant, *F*(12, 114) = 3.63, *p* < 0.001, with an adjusted *R*² of 0.20.

## Discussion

This study investigated caries experience and associated factors in an adult Norwegian psychiatric inpatient population. The current results indicate substantial unmet treatment needs in the inpatient population. In particular, mean number of DT (4.6) was almost four times greater than in the general adult population in Norway, where mean values between 1.1 and 1.4 have been reported [40, 41]. Even the youngest participants in this study, despite having access to free or subsidized dental care until the age of 21 [27], had about 2.5 times more DT than what has been reported for young adults in the general Norwegian population [40, 41], indicating severe decay at an early age.

In the general population the caries distribution is highly skewed, where most individuals have none or only one decayed tooth [40, 41]. In contrast, 45% of the participants of this study had four or more DT, and 24% had eight or more DT. These results demonstrate a very high prevalence of active dental disease in this population and a severe disparity in oral health compared to the general population.

In this study, the mean number of FT was 7.9, ranging from 5.4 in the 19–29-year age group, to 11.7 in the 50 years or older age group. Whereas DT shows active disease and need of treatment, FT is a measure of previous, accumulated caries experience and an indirect indicator of access to dental care. Thus, the number of FT usually increases with age as DT are restored. The higher number of FT in the older age groups in this study suggests that the participants have had access to dental services, however to much less extent than needed based on the high number of DT. A recent study on a Norwegian general adult population found the mean number of FT to be 11, ranging from 3.9 in the 19–24 year age group to 14.8 in the 55–74-year age group [40]. Variations in age group classifications across different studies complicate comparisons of results, however the FT index seemed to differ less than DT between the inpatient population of this study and previous reports from the general Norwegian adult population.

Research indicates that individuals with psychiatric and/or substance use disorders visit the dentist less frequently than the general population [8, 9, 11, 42]. Consequently, oral diseases such as dental caries progress further, leading to a need for more invasive and costly treatments. Often, this results in tooth extraction rather than conservative treatment options. Thus, a significant number of MT, particularly at a younger age, implies severe dental decay or periodontal disease that has not been adequately managed. The mean number of MT for participants in this study was 2.6, which is similar to the reports from the general Norwegian population (mean 2.4–3.1 [40, 41]). However, given the relatively low mean age in the present sample, the extent of tooth loss is concerning.

Twenty remaining teeth is considered a minimum for normal oral functioning [43–45]. In this study, 13% in the 40–49-year age group, and 20% in the 50-year and older age group had less than 20 teeth.

In contrast, fewer than 1.5% of adults in the general adult Norwegian population had such an extensive tooth loss before age 60 [36]. Of note, two edentulous participants were excluded from this study, meaning that the mean number of MT is somewhat underestimated. Our findings demonstrate a deterioration of oral health at a relatively young age in patients suffering from psychiatric and substance use disorders that may disturb oral functions and contribute to overall poorer health.

While caries experience and tooth loss were substantially higher in our study population compared to the general Norwegian adult population, our findings are consistent with international research on individuals with substance use or psychiatric disorders [18, 20, 21, 46, 47]. Although variations in age range and small sample sizes in many of these studies reduce the precision of prevalence estimates, the overall trends are remarkably consistent, as demonstrated in several systematic reviews [17, 18, 46, 48]. Previous studies have also found that the extent of tooth decay was generally greater for people requiring inpatient care as well as for those with chronic and more severe psychiatric symptoms [17], where a high number of DT was often accompanied by a high number of MT rather than fillings. This cross-national consistency suggests that the oral health challenges observed in this group are not limited to a Norwegian context but reflect broader, structural patterns.

All participants in this study were inpatients treated for mental and/or substance use disorders and suffered significant illness at the time of the study. A significant association was observed between reports of substance use and DT, while no significant associations were found between mental health variables and DT. Of note, data on substance use and psychiatric illness were self-reported. Given the frequent co-occurrence of psychiatric and substance use disorders [49], disentangling their individual effects on oral health is challenging. Nonetheless, the observed pattern underscores the particularly negative role of substance use on dental outcomes within this clinical context.

Substance use, and psychiatric illnesses may cause poor oral health due to several interconnected factors. These conditions often lead to neglect of oral hygiene routines [17, 22], increased consumption of sugary foods and drinks, and higher tobacco use [15, 24]. In addition, medications used to manage mental health disorders can cause dry mouth (xerostomia) [17], increasing the risk of dental decay. Limited access to dental care combined with stigma and reduced motivation [8, 42], further exacerbates the problem. In this study just over half of the participants brushed their teeth at least twice a day, and 24% brushed less than daily. This is a much lower brushing frequency than in the general Norwegian adult population where more than 70% report brushing at least twice a day [36]. Participants who brushed less than daily had approximately 70% more DT compared to those who brushed daily or more. Toothbrushing was also significantly associated with DMFT, underscoring the importance of oral hygiene habits in maintaining oral health, which is in line with previous findings [50]. Promoting consistent oral self-care in this population may therefore be an important step toward improving oral health outcomes.

Socioeconomic factors are known to influence oral health. Mental health issues and substance use can disrupt education and employment [8, 24], leading to financial instability, and limit access to dental care. In our study, 36% of the participants had only elementary education, and less than 5% held a college or university degree, substantially lower than the general Norwegian population, where 16% of those in the 20–60-year age group have elementary school as highest completed education, and approximately 22% hold higher degrees [51].

Participants with elementary education had more DT than those with higher education, although this association was only borderline significant after adjustment. Descriptive statistics also supported this pattern, showing higher DT and lower FT among those with lower education levels. These findings suggest that educational attainment may still be a relevant indicator of caries risk in this population, likely influenced by factors such as oral hygiene, access to care, and other health-related behaviors.

Employment status also highlighted disparities as nearly 90% of participants were out of the workforce, and 40% faced financial difficulties, compared to 3.5% in the general population [37]. In this study, descriptive data showed that participants with financial difficulties had significantly more DT than those experiencing good finances. Although this association was only borderline significant in regression analysis, the trend underscores the role of both education and economic status in shaping access to and use of dental health services [8, 24], and how limited understanding or ability to maintain oral self-care may hinder timely help-seeking, often resulting in inadequate dental service use and worsening oral health outcomes [23, 52–54].

### Study’s strength and limitations

The outcome measures in this study were based on a standardized clinical examination by a trained dentist. The regression model explained approximately 28% of the variance in DMFT, which represents a large effect size for cross-sectional data in health and social sciences [55]. While this indicates moderate explanatory power, a substantial portion of the variance remains unaccounted for, likely due to unmeasured factors such as genetic predisposition, dietary habits, and environmental influences.

As a cross-sectional study, this research cannot establish causality. The sex and age distribution of our sample aligns with similar studies [20, 21, 56, 57], but as the inpatient population fluctuates, it is not possible to conclude on representativity. However, the sample size was modest, which limits the generalizability of the findings and reduces the study’s statistical power to detect smaller differences, causing a relatively low precision in prevalence estimates. In addition, the need to dichotomize or group variables may have reduced sensitivity to more subtle trends. Furthermore, the psychiatric and general medical records were not accessible for review, resulting in reliance on self-reported health variables.

There is a risk of underreporting on sensitive topics such as smoking, alcohol, and substance use [58, 59], and participants may provide socially acceptable or favorable responses [58]. Some variables from questionnaires may also be sensitive to recollection bias, as participants may have forgotten or misremember details. The questionnaire to assess symptoms of psychosis risk were originally in English, and their translation into Norwegian was not validated by forward and backward translation. Thus, subtle differences in meaning or interpretation may have affected the reliability of the responses. However, this risk was likely minimized, as the interviews were conducted by a trained assistant who could clarify any misunderstandings. The findings of this study should therefore be interpreted in light of these limitations.

## Conclusions

The findings demonstrate a high prevalence of dentine caries and significant tooth loss at a relatively young age among inpatients with substance use and/or psychiatric disorders. Compared to the general population, the participants had low socioeconomic status, and reported a low use of dental health services, inadequate oral hygiene routines, and a high prevalence of xerostomia. This study highlights the severity of oral health problems for this group of patients. It is crucial to explore how the dental healthcare system can better address the needs of this group to improve their oral health outcomes.

## Declarations

### Availability of data and materials

The datasets generated and analyzed during this study are not publicly available due to restrictions under the EU General Data Protection Regulation (GDPR), which prevents these datasets from being made into open access data. However, access to the de-identified data can be granted by the corresponding author upon reasonable request and is subject to strict adherence to privacy protocols. Interested researchers are encouraged to contact the corresponding author with a detailed proposal of their intended use of the data. All requests will undergo an ethical review to ensure compliance with relevant regulations and ethical standards.
